# Calorie intake is associated with weight gain during transition phase of nutrition in female extremely low birth weight infants

**DOI:** 10.1186/s13293-020-00295-7

**Published:** 2020-04-15

**Authors:** Pradeep Alur, Renjithkumar Kalikkot Thekkeveedu, Madaleine Meeks, Kyle C. Hart, Jagdish Desai, Marla Johnson, Sara Marie Presley, Naveed Hussain

**Affiliations:** 1 University of Mississippi Medical Center, Jackson, MS USA; 2 Connecticut Children’s Medical Center, Hartford, CT USA; 3 University of Connecticut School of Medicine, Farmington, CT USA

**Keywords:** Transition phase, ELBW infants, Parenteral nutrition, Sex difference in growth, Calories, Protein, Intralipid, Growth percentile, Preterm

## Abstract

We sought to determine whether there are sex-based differences in the requirements for calories or protein for optimal growth during the transition phase (TP) when an extremely low birth weight (ELBW) infant, defined as a preterm infant with a birth weight of < 1000 g, is progressing from parenteral to enteral feeds. A retrospective review of ELBW infants born from 2014 to 2016 was performed at a tertiary NICU. Infants with necrotizing enterocolitis, short bowel syndrome, or chromosomal anomalies were excluded. TP was defined as the period when the infant’s enteral feeds were increased from 30 up to 120 ml/kg/day while weaning parenteral nutrition (PN). Effects of sex and protein-calorie intake on the change in growth parameters from the beginning to the end of TP were analyzed. Pre-TP growth percentiles and calorie and protein intake were similar in both sexes. There was a significant (*r* = 0.22, *p* = 0.026) correlation of total calorie intake with a change in weight percentiles (wt.pc) for the whole group, but on sex-specific analysis, this correlation was more robust and significant only in girls (*r* = 0.28, *p* = 0.015). Protein intake did not correlate with the changes in wt.pc in either sex. Despite a similar intake of calories and protein during the TP, we found a significant decrease in wt.pc only in girls. More extensive studies are needed to understand the sex-based differences in caloric needs and metabolic rate in ELBW infants.

## Introduction

Optimal early nutrition in preterm infants has been associated with better growth and neurodevelopmental outcomes. The total energy intake during the first 7 days of life is positively correlated with improved growth parameters until the corrected age of 2 years [1]. Coviello et al. found a positive relationship between the initial 4 weeks of nutrition, with neurodevelopment, and white matter maturation at term equivalent age (TEA) in preterm infants < 31 weeks of gestation [2]. Higher energy and lipid intake during the first 2 weeks after birth were associated with a lower incidence of magnetic resonance imaging abnormalities of the brain at TEA in preterm infants [3].

The transition phase (TP) of nutrition is an important stage in the progression towards optimal nutrition in ELBW infants [4, 5]. All ELBW infants are initially started on parenteral nutrition (PN). Enteral feedings are gradually increased as PN is gradually reduced until enteral nutrition is the sole source of nutrition. The TP is typically completed in the first 2 weeks of life in ELBW infants. Even though it is recognized that nutrition during the TP is critically important for ELBW infants, no formal guidelines specific to the TP currently exists. In the two studies focused on TP nutrition, the effect of sex differences was not evaluated [4, 5]. Moreover, there are wide variations in nutritional practices relating to advances in fluid volume, feed fortification, and nutritional supplementation during this time.

The evidence that the establishment of early nutrition in preterm infants is associated with sex-specific differences in the outcomes is still evolving. A recent study showed that higher amino acid intake during the first week of life in preterm infants was associated with greater weight gain during the first 5 weeks of life in males compared to females [6]. Interestingly, in these infants at 2 years of age, the mental developmental index was higher in females, and the psychomotor developmental index was higher in males.

Sex-related differences in anthropometric parameters and growth are well established in infants and adults. Postnatal growth characteristics are also sex-specific; hence, distinct anthropometric standards for male and female preterm infants, such as Fenton growth chart 2013, are needed [7]. One might surmise that if the growth characteristics are distinctively sex-specific, then the caloric and protein requirements could also be sex-specific in ELBW infants. Surprisingly, there are no studies which investigate the sex differences in nutrition requirements in human ELBW infants. Moreover, it is still not clear if these growth differences are due to the differences in nutritional intake between the sexes or if there is sex variation in the metabolism of the nutrients. These differences may also be due to a combination of factors. Our current study was designed to evaluate if there were sex-based differences in growth (weight gain and head growth ) based on the effect of protein and calorie intake during the TP of nutrition in ELBW infants.

## Materials and methods

### Study population

The study design was a retrospective cohort analysis of ELBW infants born from 2014 to 2016 at a level 4 NICU at the University of Mississippi Medical Center (UMMC). The study was conducted in accordance with the Declaration of Helsinki, and the protocol was approved by the Ethics Committee of UMMC and was approved by the UMMC institutional review board on August 16, 2017 (IRB file# 2017-0120). The inclusion criterion was ELBW infants born between the years 2014 and 2016 at UMMC. The exclusion criteria were (1) Infants with short bowel syndrome and/or dependence on total PN, (2) infants who were nothing per os (NPO) for 10 days or more in the first 30 days of life, (3) infants with severe chromosomal anomalies known to interfere with postnatal growth and survival, (4) infants with prostaglandin-dependent cardiac lesions, (5) infants with hydrocephalus, and (6) infants diagnosed with necrotizing enterocolitis (NEC).

There were 123 ELBW infants that remained in the study after exclusion. Of these, there were 28 (22.7%) small for gestational age infants who were excluded from the analyses as their growth rates were expected to be very different from appropriately grown ELBW infants. We analyzed 95 ELBW infants, 59 females and 36 males, who were appropriate for gestational age at birth in our study cohort.

Data related to the following variables were collected from a review of databases and patient charts: demographic data including gestational age, birth weight and head circumference percentiles, sex, and race were obtained. Nutritional data such as type of enteral feeding (mother’s breast milk, donor’s breast milk, or formula), caloric density (calories/oz) during TP, weaning, discontinuation of intralipids, mean calories/kg/day and protein in g/kg/day during TP and enteral phase, day of life birth weight regained, day of life TP initiated, and the duration of the TP were collected. Growth data included weight and head circumference percentiles, growth velocity, and *z* scores before, during, and after the TP. The following comorbidities were evaluated as potential confounders—chronic lung disease, culture-positive infections, retinopathy of prematurity, intraventricular hemorrhages, use of postnatal steroids, and patent ductus arteriosus.

### Nutritional management

#### NICU feeding protocol

All ELBW infants were started on a total daily fluid intake of 100 ml/kg/day that included 4 g/kg/day of protein as parenteral nutrition after birth. Dextrose was initiated as 7.5 to 10% in PN to provide an initial glucose infusion rate of 4 to 6 mg/kg/min and subsequently increased to provide a glucose infusion rate of up to 11 mg/kg/min. The current standard of care for parenteral nutrition fluids as recommended by the American Academy of Pediatrics includes vitamins, electrolytes, and trace elements in standard doses for all ELBW infants [8]. Intralipids were started at 1 g/kg/day and were advanced as tolerated to give a maximum of 3 g/kg/day while maintaining the serum triglyceride level below 200 mg/dl. Enteral nutrition was started with human milk either the mother’s own milk or donor’s breastmilk and maintained as trophic feeds (10 to 30 ml/kg/day) for 1–3 days after birth before beginning the TP of nutrition. Donor breast milk used in the USA is currently pooled milk from all the mothers irrespective of the sex of their offspring.

#### The transition phase of nutrition

TP was defined as the period when the infant’s enteral feeds were increased from 30 ml/kg/day up to 120 ml/kg/day while PN was gradually reduced, with a goal total protein intake of 3.5 to 4 g/kg/day and calorie 100 to 120 kcal/kg/day. Human milk feeds were gradually increased by 10 to 20 ml/kg/day as tolerated. PN was correspondingly reduced when enteral feeds were increased to > 50 ml/kg/day to give at least 150 ml/kg/day of total fluids. Human milk was fortified to 24 cal/oz with human milk fortifier (Enfamil® Human Milk Fortifier Powder) when the volume of the enteral feed was > 50 ml/kg/day. Human milk fortification optimizes caloric content, protein, fat, vitamins, and various other micronutrients [9]. Intralipids were discontinued once the enteral feeds were greater than 80 ml/kg/day. Full enteral nutrition was defined as enteral feed volumes of more than 120 ml/kg/day without parenteral nutrition.

#### Calculation of nutritional intake

The nutritional composition of the PN and enteral feeds were obtained from the nutritional calculator within EPIC’s electronic medical record. A daily measure of total calories and protein from both PN and enteral nutrition was obtained for each ELBW infant. A reference standard was used for calculating calories and protein in breast milk [10].

#### Measurement of growth

Fenton 2013 sex-specific growth charts were used for determining the percentiles and *z* scores for weight and head circumference [7].

### Definition of terms

Chronic lung disease was defined as oxygen requirements at 36 weeks of corrected gestational age. Retinopathy of prematurity was defined as any stage of retinopathy from stages 1 to 4 diagnosed by the pediatric ophthalmologist. Intraventricular hemorrhage (IVH) was defined as any grade of IVH from 1 to 4 as diagnosed by the radiologist with a head ultrasound. Infection was defined as any culture-positive infection from blood, urine, or cerebrospinal fluid specimens. Patent ductus arteriosus was defined as the presence of a patent ductus arteriosus on an echocardiogram as diagnosed by the pediatric cardiologist.

### Statistical analyses

Univariate analyses using independent *t* tests for continuous variables and chi-square test for nominal variables were done. Non-parametric tests were done for samples that did not show normal distribution. We performed the Pearson correlation tests in males and females to evaluate the association of weight change during the TP for the given number of calories and protein. Subsequently, multivariable backward logistic regression analysis was done to confirm this correlation in males and females while accounting for the effect of potential confounders. We used TP calories, protein, duration, and day of life of TP initiation along with the weight percentile before TP as confounders. Statistical significance was determined for inferential statistical tests when *p* value was ≤ 0.05.

## Results

### Study population

Of the 123 ELBW infants that remained in the study after the initial exclusions, there were 28 (22.7%) small for gestational age infants who were further excluded from the analyses as their growth rates were expected to be very different from appropriately grown ELBW infants. The final analysis was based on a total of 95 ELBW infants, 59 females and 36 males, who were appropriate for gestational age at birth in our study cohort.

Sex-specific anthropometric data of the study population is shown in Table 1.

**Table 1:** Cohort characteristics

Cohort characteristics (*N* = 95)	Characteristics, mean ± SD or *N* (%)		*p* value*
	Male (*N* = 36)	Female (*N* = 59)	
Demographic characteristics			
Gestational age (weeks)	26.4 ± 1.7	26.2 ± 1.7	0.52
Race: African-Americans	31 (86%)	47 (80%)	0.82
Anthropometric data			
Weight			
Birth weight (g)	856 ± 110	789 ± 153	0.02
Birthweight centile	43 ± 25	41 ± 21	0.65
Mean weight centile before the transition phase	21 ± 13	25 ± 16	0.14
Mean weight centile after the transition phase	21 ± 12	23 ± 14	0.44
Mean weight centile change (before the transition phase-after transition phase)	+ 0.19 ± 8.6	− 2.2 ± 8.2	0.17
Head circumference			
Birth HC centile	24 ± 1.6	23 ± 1.6	0.26
Mean HC centile before the transition phase	16 ± 19	21 ± 20	0.22
Mean HC centile after the transition phase	14 ± 19	17 ± 15	0.57
Mean HC centile change (before the transition phase-after transition phase)	− 1.08 ± 13	− 3 ± 14	0.51
Comorbidities			
Chronic lung disease	22 (61.1)	32 (54.2)	0.53
Patent ductus arteriosus	17 (47.2%)	31 (52.5%)	0.67
Any intraventricular hemorrhage	12 (33.3%)	17 (29%)	0.64
Any retinopathy of prematurity	11 (30.6%)	27 (45.8%)	0.19
Culture positive infection	4 (11.1%)	13 (22.0%)	0.27
Postnatal steroid	8 (22.2)	15 (25.4)	0.8

*Chi-square test for a categorical variable and independent sample *t* test for continuous variables

Table 1 also shows that the comorbidities of chronic lung disease, patent ductus arteriosus, intraventricular hemorrhage, sepsis, postnatal steroids, and retinopathy of prematurity did not differ between sexes.

As expected, females weighed significantly less than males, and the percentiles of birth weight, length, and head circumference based on the sex-specific Fenton growth charts did not differ between sexes.

### Nutritional characteristics

Sex-specific nutritional characteristics of the study population are shown in Table 2. It shows that the average daily calorie intake (103.6 ± 7.2 in males versus 102.4 ± 7.7 kcal/kg/day in females, *p* = 0.46) and protein intake (3.8 ± 0.44 in males versus 3.7 ± 0.53 in females, *p* = 0.75), were not different between the sexes.

**Table 2:** Nutritional characteristics

Nutrition characteristics (*N* = 95)	Characteristics, mean ± SD or N (%)		*p* value*
	Male (*N* = 36)	Female (*N* = 59)	
Mean calories (kcal/kg)	103.6 ± 7.2	102.4 ± 7.7	0.46
Mean protein (g/kg)	3.8 ± 0.44	3.7 ± 0.53	0.75
Maternal breastmilk usage	19 (57.5)	40 (67.7)	0.32
Feeding volume fortified to 24 kcal/oz	88 ± 33	94 ± 34	0.42
Day of life transition phase began	11 ± 8	10 ± 6	0.35
Duration of the transition phase	10 ± 3	9 ± 3	0.49
Day of life birthweight regained	9 ± 6	8 ± 6	0.75

*Chi-square test for a categorical variable and independent sample *t* test/Mann Whitney *U* test for continuous variables

There was a significant (*r* = 0.22, *p* = 0.026) correlation of total calorie intake with a change in weight percentiles for the whole group. The pre-TP growth percentiles did not differ between sexes.

**Fig. 1:**
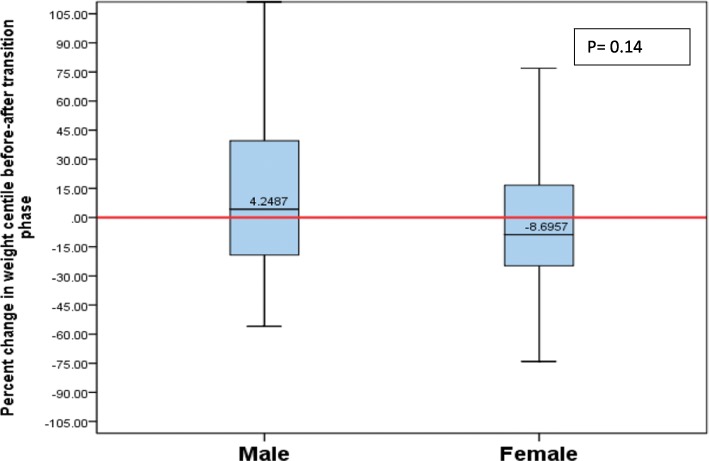
Percentage of change in weight percentile from pre-TP to post-TP by sex

**Fig. 2:**
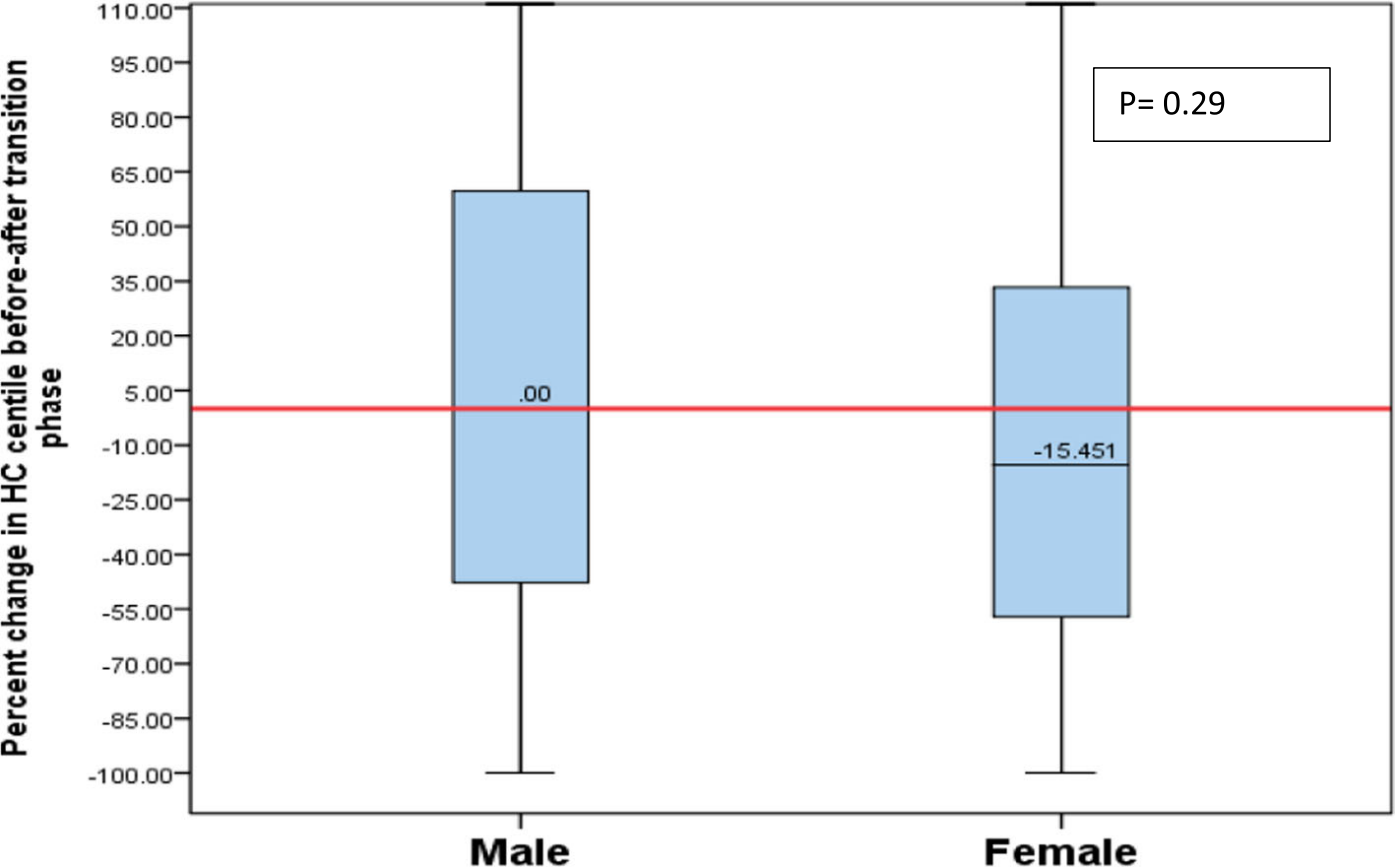
Percentage of change in head circumference percentile from pre-TP to post-TP by sex

Overall, girls lost 8.7% of their weight percentiles, whereas boys gained 4.24% of weight percentiles from pre-to-post-TP (*p* = 0.14) (Fig. 1). However, this did not reach statistical significance indicative of no sex difference. Girls had a decline of 15.45% of head circumference percentiles, while boys maintained their head circumference percentiles from pre-to-post-TP, and these results, however, were not statistically significant (*p* = 0.29) (Fig. 2).

**Fig. 3:**
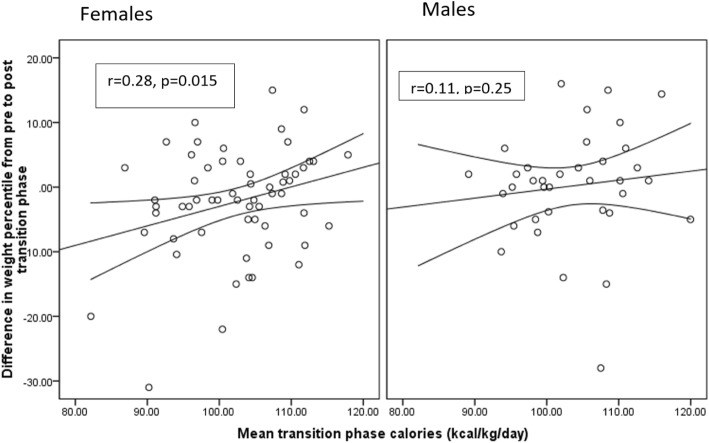
Correlation between total calorie intake and change in weight percentiles

However, on sex-specific analysis, total calorie intake significantly correlated with a change in weight percentiles only in girls (*r* = 0.28, *p* = 0.015) (Fig. 3). This correlation was further evaluated by backward regression to account for the effect of confounders. Final predictors of percentile weight change during TP in females using a backward linear regression model were calorie intake during TP and weight centile before TP. In males, this correlation remained statistically non-significant in the regression model.

Correlation of protein intake and head circumference change during TP in males (*r* = − 0.15, *p* = 0.23) and females (*r* = − 0.098, *p* = 0.56) was not statistically significant. Similarly, the correlation between protein intake and weight change during TP in males (*r* = 0.017, *p* = 0.89) and females (*r* = 0.006, *p* = 0.97) was not statistically significant.

## Discussion

Our study shows that despite similar calorie intake during the TP, girls’ weight percentiles were more affected by caloric intake compared to boys. This finding remained significant even after adjusting for potential confounders. Future studies including a larger cohort may provide confirmation as to whether ELBW girls are more susceptible to weight loss compared to ELBW boys. The state of nutrition before the beginning of the TP was not a confounding factor as the mean weight and head circumference percentiles before TP were similar for both sexes. Duration of the TP, day of life for regaining birth weight, day of life of starting TP, mother’s own milk usage, chronic lung disease, and postnatal steroid use were also similar between girls and boys. These data support the hypothesis that nutritional requirements could be different between preterm boys and girls during the TP.

Sex differences in metabolism and growth are well-recognized, starting from conception and lasting through all stages of life [11]. Crown-rump length and biparietal diameter in human male fetuses are larger than in females starting at the first measurement between the 8th and 12th weeks [12]. Even postnatal growth characteristics are sex-specific; hence, distinct anthropometric standards for male and female preterm infants, such as Fenton growth chart 2013, are needed [7]. One might deduce that if the growth characteristics are sex-specific, then the caloric and protein requirements could also be sex-specific in ELBW infants. Surprisingly, there are no studies which investigate the sex differences in nutrition requirements in human ELBW infants, especially during the early phase of the establishment of enteral nutrition.

There is some evidence from animal studies that nutrition requirements may be sex-specific in the first few months of life. Animal milk volume and composition differ based on the sex of the offspring. Cows produce more volume if the offspring are female [13]. Similarly, primates such as rhesus macaque monkeys produce higher calcium content milk for daughters and energy-rich milk for son [14, 15]. In summary, natural biological processes in animals provide milk with distinctively different compositions to suit the nutritional needs based on the sex of the offspring. On the other hand, the available evidence of sex-specific differences in human milk composition are not well established [16–21].

Current evidence in preterm infants suggests that not only is somatic growth different between the sexes, but body composition differs as well. The intergrowth-21 study concluded that fat mass was higher in girls than boys, even in preterm infants [22]. Lean mass is higher in male preterm infants compared to female preterm infants. Since nutritional intake and body composition are closely related, one can argue that the nutritional requirements of preterm male and female infants may also be different when their body compositions are distinct.

Studies have indicated that early nutrition in very low birth weight (VLBW) infants can have sex-specific outcomes. A deficit of fat-free mass in preterm infants at discharge is associated with neurological impairment at 2 years of age [23]. Poindexter showed that lower intake of amino acids early in life was associated with a smaller head circumference at 18 months of age only in males [24]. Recently, a European study demonstrated that higher amino acid intake during the first week of life in preterm infants was associated with higher weight gain during the first 5 weeks of life only in males [6]. This study also reported that the mental developmental index was higher in girls, and the psychomotor developmental index was higher in males at 2 years of age. Van den Akker et al. showed that VLBW boys had a better outcome (survival without neurodevelopmental disabilities) significantly more often than girls if amino acids were administered very early after birth [25]. Hence, appropriate nutrition may need to be provided in a sex-specific manner to achieve optimal body composition and improve long-term outcomes.

Our study results are confined to the TP of nutrition and growth outcomes during the TP. In contrast, other studies have focused on the higher protein (2-3 g/kg/d) versus lower protein (2 g/kg/day) provided in the parenteral nutrition and evaluate for long-term outcomes. Another distinct feature in our cohort of infants was that they received 4 g/kg/day of protein immediately after birth in their parenteral nutrition fluids. To our knowledge, there are no sex-specific short-term growth outcome studies during TP of nutrition, and our study results may provide unique preliminary findings.

We consider our study precursory in reporting sex-based differences in the growth effect of protein and calorie intake during the TP of nutrition in ELBW infants. The strength of our study is that the nutritional management of all infants was standardized based on well-enforced unit guidelines. Breast milk fortification between the groups was similar, which reduces the possibility of micronutrient intake differences between the sexes. Our study provides preliminary evidence that nutritional requirements may be different between the sexes in preterm infants during the TP of nutrition. The main limitations of our study are that this is a single-center retrospective pilot study and that it is based on a relatively small sample size. Comorbidities such as patent ductus arteriosus, postnatal steroids, and intraventricular hemorrhage may affect anthropometric measurements during the TP of growth, but there is no current evidence in the literature on this subject. However, in this current study, the comorbidities were not significantly different between sexes. Moreover, the initial respiratory status, ventilatory support, or the severity of illness in the first few days of life were also not available for analyses. Nevertheless, these are the prime factors for the occurrence of chronic lung disease and the use of postnatal steroids after the transition phase is complete in this population. As the incidence of chronic lung disease and the use of postnatal steroids are not different between the sexes (Table 1), the above limitations may not be significant. Future studies can provide confirmation. Our results may be most relevant in formulating a larger prospective study with an appropriate sample size and power to confirm our findings. Prospective nutritional and metabolic studies are needed to understand sex-specific nutritional requirements in preterm infants and the optimal composition of nutrients necessary to promote physiological body composition characteristics.

## Conclusions

Despite similar intake of calories and protein during the TP of nutrition in ELBW infants, there was a significant correlation of caloric intake with weight percentile changes only in girls but not in boys. Larger prospective studies are needed to confirm our findings, as well as to understand the biochemical and physiological basis for such sex-related differences.

## Data Availability

We the authors agree for the data to be deposited in a publicly accessible repository, once the manuscript is accepted for publication.
